# Using a Knowledge and Awareness Survey to Engage and Inform a Community-Based Tuberculosis Intervention among Nomads in Adamawa State, Nigeria

**DOI:** 10.3390/tropicalmed9080167

**Published:** 2024-07-23

**Authors:** Stephen John, Suraj Abdulkarim, Thandi Katlholo, Caoimhe Smyth, Hunpiya Basason, Md. Toufiq Rahman, Jacob Creswell

**Affiliations:** 1 Janna Health Foundation, Yola 640231, Nigeria; wizemannstv2@gmail.com (S.J.); hunpies@yahoo.com (H.B.); 2 SUFABEL Community Development Initiative, Gombe 760253, Nigeria; drsurajkwami@gmail.com; 3 Country and Community Support for Impact Team, Stop TB Partnership, 1218 Geneva, Switzerland; thandik@stoptb.org (T.K.); caoimhes@stoptb.org (C.S.); 4 Innovations & Grants Team, Stop TB Partnership, 1218 Geneva, Switzerland; toufiqr@stoptb.org

**Keywords:** KAP, nomads, tuberculosis, active case finding, community engagement, key populations

## Abstract

Background: Nomadic populations are frequently isolated and vulnerable to diseases including tuberculosis (TB) and human immunodeficiency virus (HIV) due to limited access to health-related information and services, poverty, and social exclusion. We designed and implemented community-driven and -based outreach for TB and HIV based on the results of a TB knowledge, attitude, and practices (KAP) survey in Adamawa, Nigeria. Methods: We conducted a cross-sectional study on KAP among nomads using an adapted WHO survey. A TB and HIV community-level active case-finding intervention among nomadic populations was planned and delivered based on the KAP survey results. Results: Among 81 respondents, 26 (32.1%) knew what caused TB. More than 60% reported no health facilities in their community. Radio and healthcare workers were primary sources of information on health. Using community input, we developed and broadcasted radio jingles to sensitize people to TB services. Outreach initiatives led to the verbal screening of 61,891 individuals and 306 were diagnosed with TB. Additionally, 4489 people underwent HIV testing, and 69 were HIV-positive, all of whom were linked to treatment. Conclusions: The results of KAP surveys can inform the design of evidence-based TB and HIV community-driven and -based case-finding interventions in rural Nigeria among nomadic populations.

## 1. Introduction

With an estimated 452,000 people who develop tuberculosis (TB) and 156,000 who die from the disease annually, Nigeria ranks fifth among the 30 high TB burden countries, and it has by far the highest TB burden in Africa [1]. Even though TB is curable, it is the second leading cause of death from an infectious disease globally after COVID-19 [1]. Reasons for this include suboptimal treatment-seeking behaviour, low case detection, delayed diagnosis, poor treatment adherence [2,3], limited access to health services, disease-based discrimination, lack of privacy protections, and the impact of patriarchal norms on women affected by TB [4]. Similarly, human immunodeficiency virus (HIV) infection is one of the world’s most significant health, human rights, and development challenges and the leading risk factor for TB [5]. In several Sub-Saharan African countries, two-thirds of people who develop TB each year are also HIV-positive, while one-third of people who die of TB are HIV-positive [1]. TB and HIV are both among the top six causes of death in Nigeria [6].

While TB and HIV services are widely available in most high-burden countries, globally, more than four million people do not receive proper TB diagnosis and treatment each year, and Nigeria alone accounts for more than 7% of the global figure [1]. Globally, about six million people were unaware that they were HIV-positive in 2020. Many people who are missed by routine health services are key populations as they are at increased risk of contracting TB or have poor access to TB care [7,8]. In Nigeria, nomadic populations are especially prone to TB and HIV. Nomads are communities of people who constantly migrate in search of pasture for their livestock, subsisting on hunting and gathering or often driven by climatic conditions [9,10]. Nomadic populations are often isolated and are socially vulnerable to diseases such as TB and HIV infections due to limited access to health-related information and health services, economic poverty, the use of traditional/alternative medicine, geographical isolation, social exclusion, and migration [9,11,12]. Significant gaps exist in awareness and practices related to TB prevention and treatment among nomadic populations, and involving community leaders in health interventions has been shown to enhance both acceptance and effectiveness [13]. Estimates of the nomadic population are wide, maybe 50–100 million people globally, including 9.4 million in Nigeria [14].

Documenting gaps in knowledge, attitudes, and practices (KAP) regarding TB and proposing solutions is critical to the End TB transformative agenda. Adequate knowledge about TB is a powerful means of empowering people affected by TB, promoting health-seeking behaviour and equitable access to quality treatment and prevention programmes. Thus, KAP surveys reveal gaps in and barriers to access and violations of the right to health for TB-affected individuals and their communities. This publication demonstrates the commitment of the Janna Health Foundation (JHF) as a duty bearer towards the fulfilment of the right to health through the promotion and provision of health-related research [15].

While designing community-based interventions, community engagement and understanding the knowledge, perceptions, and practices of the population are critical. The Stop TB Partnership produced a guide on how to conduct KAP surveys as part of the development of community-based activities to address TB [16]. Over the last ten years, many KAP surveys on TB have been conducted in different settings [17,18].

In Nigeria, there have been several KAP surveys published. These have focused on community members, people with TB, and health workers [19,20,21]. Often, KAP surveys are reported as standalone studies, and the results are not interpreted from a people-centred perspective. One such study from Nigeria involved KAP results from only one TB-affected person, which were subsequently used to inform a full TB intervention [22]. We could not identify publications engaging key populations, and KAP among these groups in TB may be quite limited, unlike the wealth of information in the HIV community among key populations [23,24]. 

Engaging and understanding the knowledge and perceptions of key populations is essential to design appropriate interventions that meet their needs. Here, we present the results of a KAP survey of Nigerian nomads on TB and HIV in Adamawa State, which informed the design of a TB and HIV community-based case-finding intervention that addressed several identified barriers.

## 2. Materials and Methods

### 2.1. Study Setting

Adamawa State is located in the northeastern part of Nigeria with an estimated population of 4.3 million (projected from the 2006 National Census) [16], about 10% of whom are nomadic people. The study area included 12 intervention Local Government Areas (LGAs), which are similar to districts in other countries, containing 628 health facilities (both public and private). Of the total facilities, 109 provided TB treatment services, while 32 provided diagnostic services. There were four Xpert MTB/RIF testing sites in the study area (Numan, Ganye, Yola, and Mubi) during implementation. Each diagnosis and treatment unit used standard recording and reporting tools issued by the National Tuberculosis and Leprosy Control Programme (NTBLCP). Among all facilities, 137 delivered HIV testing services (HTS), including 41 that provided services for the prevention of mother-to-child transmission of HIV, while 9 provided comprehensive ART services.

#### Study Design

The study consisted of two phases: a pre-intervention phase and an intervention phase. 

### 2.2. Pre-Intervention Phase

#### 2.2.1. KAP Survey

A cross-sectional study of the knowledge, attitudes, and practices of TB among the nomads in Adamawa State was conducted in May 2018. A multistage sampling technique was employed to recruit participants. Stage 1: of the 12 LGAs where the active case finding (ACF) was planned, three LGAs were purposefully selected for the KAP survey based on the numbers of nomadic schools and host communities obtained in a master list from government officials involved with Nomadic Education. Stage 2: in each of the three LGAs, three nomadic schools were then randomly selected from a master list. Additionally, nomadic communities around each school were randomly selected for inclusion in the KAP survey. Stage 3: Four teachers were randomly selected from the school’s teacher employment roll from each selected school. The targeted number of teachers was divided by the total number of teachers in the employment role to obtain an interval that guided the selection from the first name which was identified by blind-pointing. Subsequent numbers were picked using the interval until all the teachers were identified. The selection of community leaders was done purposively, as they are recognized by their communities as guardians and representatives. For the selection of community members, we employed a random sampling technique. A specific number of community members were allocated for selection in each chosen community. Using the household of the community leader as the central point, houses were grouped into four clusters extending in different directions. Every third house in each cluster was selected. From each selected house, an adult who met the pre-determined criteria (consent, age [18–60], sane mind, and physically fit) was chosen for an interview. If the selected individual did not meet the criteria, the next house was selected. After discussions with community leaders, five individuals (two community leaders and three community members) were invited to participate. Thus, the study population was inclusive and consisted of three groups (nomadic teachers in primary schools and community leaders as well as members from nearby nomadic communities). After obtaining written and verbal informed consent, participants were interviewed. 

#### 2.2.2. Data Collection Methods

Data were collected using a structured interviewer-administered questionnaire, which was adapted from the World Health Organization (WHO) sample advocacy, communication, and social mobilization (ACSM) KAP survey questionnaire (Supplementary File S1) [25]. The questionnaire was pre-tested in a similar community outside of the study sites by conducting 10 to 15 mock interviews, and appropriate amendments were made. The survey collected information on personal characteristics, TB knowledge and awareness, attitudes, care-seeking behaviour, and stigma. Knowledge was assessed by a series of five multiple-choice questions about TB and HIV/AIDS, attitudes were measured by four questions, and practices about access to care and from whom people received their information about the diseases were measured using six questions. 

#### 2.2.3. Interview Procedure

Face-to-face interviews were conducted by six trained research assistants with post-secondary education and fluency in the local languages. Six interviewers with required language skills were trained both through lectures and an interactive practical approach. Interviews were conducted at school or during home visits. Participants were allowed to choose comfortable locations and speak the language convenient to them, but the analysis was carried out in English. Fieldwork supervision to ensure that privacy, confidentiality, interviewing techniques, and sampling methodologies are adhered to was conducted by the project team through spot checks during the study.

### 2.3. Intervention Phase

#### ACF Intervention

Based on the findings of the KAP survey, gaps in knowledge of TB and HIV, negative perceptions about HIV, and misconceptions rooted in the nomadic culture were documented while interventions aligned with these findings were designed and implemented in the TB and HIV community-based intervention engaging nomadic populations to improve detection from 1 July 2018 to 30 June 2019. The main findings around detection and treatment are presented in the results section. This was carried out after a targeted and accessible media campaign, developed in collaboration with community members through a series of community meetings. The media campaign was developed in local languages highlighting the dangers of TB and HIV/AIDS, the signs and symptoms, how to protect people against them, and how to get the proper care. 

In collaboration with the state and LGA TB and leprosy control teams, a total of 139 nomadic schools and 128 nomadic communities from 12 LGAs with the highest population of nomads in Adamawa state were mapped and targeted for ACF. Nomadic community leaders were identified and engaged. With the support of the community leaders, 160 volunteers made up of nomadic school teachers and youths from the nomadic communities, 15 Health Care Workers (HCWs), including five Medical Officers (MOs) and five laboratory staff from TB diagnostic sites, were identified and trained on TB and HIV/AIDS basics, symptoms recognition, patient counselling, and the referral system. A typical TB screening day was preceded by a joint advocacy visit by the project staff with representatives of the state and LGA TB teams; a date and time was then agreed upon with the nomadic community leaders. TB screening was performed by community volunteers identified from the nomadic community using a verbal questionnaire in a house-to-house approach—asking for prolonged cough among adults and older (>6 years) children, while additional symptoms such as stunted growth, fever, and swelling around the neck and back were sought among children <6 years. To ensure that women and girls were equally screened for TB, female volunteers were allowed by nomadic leaders and household heads to screen women and girls in their households. Anyone who was identified with presumptive TB (persistent cough for two weeks or more, weight loss, night sweats, and fever) was asked to submit a spot sputum specimen which was transported by a designated volunteer to the nearest TB diagnostic facility for testing with Xpert MTB/RIF in line with the national guidelines. The Xpert MTB/RIF assay is a cartridge-based nucleic acid amplification test that works with the GeneXpert System. Children aged <6 years with presumptive TB were transported to health facilities where possible for further TB screening by MOs. Results from the Xpert MTB/RIF were retrieved by designated volunteers to the community. In addition, adults with presumptive TB were offered HIV counselling and testing (HCT). People with positive Xpert test results were linked to care and those unable to produce sputum and with negative results were referred for clinical evaluation and management. People diagnosed with TB and HIV were actively linked to treatment by trained community volunteers. People with TB were placed on standardized regimens based on the NTP guidelines. 

### 2.4. Data Analysis

The training and pre-testing of tools were conducted to ensure consistency and compliance with the study protocol and ethics. Data were exported to SPSS version 20.0 statistical software for analysis, and descriptive statistics were analysed. For the ACF intervention, we followed the TB screening cascade developed by TB REACH [26], documenting the numbers of people screened, people with presumptive TB, tested, diagnosed, and registered for treatment along with the relevant indicators of the number needed to screen (NNS) and the number needed to test (NNT) to identify a person with TB.

### 2.5. Ethical Approval

Ethics approval for the study was obtained from the Scientific and Research Ethics Committee of the Adamawa State Ministry of Health. Verbal informed consent was obtained, as many participants could not read or write. In addition, permission to conduct the intervention was sought and obtained from nomadic community leaders from all participating communities. Authors had access to information that could identify individual participants during data collection. The KAP study was implemented with support from the Stop TB Partnership Challenge Facility for Civil Society (CFCS) (Round 8), and the subsequent active TB case-finding intervention was implemented through the Stop TB Partnership CFCS and TB REACH funding.

## 3. Results

In total, we included 81 individuals for interviews, and all 81 (100%) agreed to participate. Among them, 36 were teachers from nine schools, 18 were community leaders, and 27 were community members. The parameters of age, sex, educational level, and occupation of the participants in the pre-intervention phase are presented in Table 1. The median age of the respondents was 40.5 years, and the majority, 62 (76.5%), were male. Only 17 (20.9%) had completed high school, while 18 (22.2%) had not had any formal education at all. Just over half, 43 (53.1%), reported working with cattle rearing or in crop farming, while 20 (24.7%) worked in the government sector.

### 3.1. KAP Survey

Findings from the KAP survey are presented in Table 2. Overall, 61.7% of the respondents reported ever hearing about TB. Nomadic teachers were more likely to report hearing about TB (69.4%), followed by community leaders (61.1%) and community members (51.9%). Overall, many gaps in knowledge were documented. Only one-third (32.1%) of all respondents knew what caused TB, ranging from 41.7% among teachers to 14.8% among community members. Fever (37%) and cough (29.6%) were the signs of TB most commonly identified. Only 13.6% said that TB and HIV/AIDS complemented each other, and a greater proportion of respondents (18.5%) said they were the same. 

Overall, a similar proportion of respondents reported hearing about TB (61.7%) and HIV/AIDS (60.5%), with high rates among teachers (72.2%). More than half (55.6%) of the participants perceived HIV/AIDS as a very serious disease, although 24.7% considered it as not very serious, and 34.6% stated that it is curable. 

A summary of healthcare and access-related findings is presented in Table 3. Radio was the clear choice of media broadcast that was used to gain information on TB, but respondents also mentioned talking to health care workers and family, friends, neighbours, and colleagues as important sources of information. The lack of any health facility in the community was reported by over 55% of the participants. A total of 37% of the study population had to travel 1 to 2 km to access the nearest health facility, while 14.8% had to travel more than 5 km. The majority lived closest to a health centre (75.3%), while slightly less than one in five individuals reported living near a hospital. 

### 3.2. ACF Intervention

A lack of knowledge about TB and HIV/AIDS, coupled with access barriers to care and support, highlighted the need for interventions designed by and for nomadic communities. Since respondents reported receiving health information from the radio and directly from HCWs, an intervention was designed with community members to provide community-based outreach services leveraging trained volunteers from the nomadic communities and HCWs for TB and HIV testing. 

Overall, 80 slots of radio jingles (two slots per week) were broadcast in Fulfulde, the nomads’ local language. These jingles covered essential messages such as TB being caused by a germ and not an evil spirit, its airborne transmission, the symptoms of TB, and the importance of seeking medical attention if coughing for more than two weeks. We emphasized that diagnosis and treatment are free and effective, traditional medicines cannot cure TB, and the need for good ventilation to prevent the spread of TB. Additionally, we highlighted that TB can also infect cattle and can be transmitted through unpasteurized milk. Furthermore, the project hosted a total of 10 meetings between LGA TB supervisors, the project team, and the state TB project team. Besides several meetings with Nomadic Community leaders, which preceded each of the 128 Nomadic community TB screenings, two meetings were held with 25 key stakeholders on the project during the project period. 

#### Nomadic School and Community Screening Events for TB

The volunteers conducted active TB screening in 139 nomadic schools and 128 nomadic communities. Overall, 61,891 verbal screening encounters for TB among nomads were documented (Table 4); 30,535 (49.3%) were adult females, 3507 (5.7%) were <5 children, while 11,043 (17.8%) were children aged 5–14 years. Of all individuals verbally screened, 5093 (8.2%) were found to have presumptive TB, while 4547 (88%) of them had their sputum collected and transported for testing with Xpert. Laboratory testing identified 225 people with bacteriologically positive TB. None of the Xpert tests identified rifampicin resistance. Of people with presumptive TB but who could not produce sputum or with negative Xpert results, 81 were started on treatment based on clinical findings for a total TB prevalence of 494/100,000 among people screened. Of all the people with TB detected, 39 were children (<15 years) and 31 of them had bacteriologically positive TB. 

Of those with presumptive TB, 4489 (88%) were tested for HIV. More females were tested (2352, 89%) than males (2137, 87%). A total of 69 (1.5% of those tested) nomads were found to be HIV+, of whom 48 were males. All individuals found to be HIV+ were linked to ART sites for further treatment, care, and support. TB cases notified from the nomadic schools and host communities through this project also had HCT at the treatment facilities; no case of TB/HIV co-infection was detected.

## 4. Discussion

The present study assessed knowledge, attitudes, and practices toward TB and HIV among Nomads in Adamawa, northeast Nigeria, and subsequently used the findings to design a community-driven and -based case-finding intervention. Similar to the Nigerian Community, Rights, and Gender Assessment findings [4] and several other studies [16,27], our study highlighted significant gaps in knowledge about TB and HIV, combined with challenges in accessing care. We also found that radio broadcasts were the main source of information on TB for many people and the second most reported source of information on HIV, findings that are similar to what was documented earlier [28,29]. To address the prevalent gaps in disease understanding among nomads, local media campaigns were provided using the local language (Fulfulde), especially considering the low literacy rates. The limited access to diagnostic and treatment services is a problem, especially in northeast Nigeria, and is exacerbated by the destruction of health facilities by Boko Haram [30] and the nomadic lifestyle and distrust of government services by many nomads [9]. This concern was further amplified by our findings.

While the results of KAP surveys are recommended to be used in improving health programming and interventions for the communities involved [25], these are rarely described in the published literature as part of TB interventions. We used the findings from the KAP survey to implement a community-based intervention for TB and HIV among the highly marginalized nomadic groups in northeast Nigeria. Our intervention was designed with communities and used radio jingles in local languages. These information campaigns were conducted collaboratively to empower people with information and increase the acceptability and uptake of health services to improve access to TB care coupled with sputum transport and sensitive molecular testing. Future strategies should include regular follow-ups and continuous monitoring to assess the medium- to long-term effects on TB and HIV. Developing culturally tailored curricula that align with the nomadic lifestyle will ensure that health education messages are effective and sustainable in promoting TB and HIV prevention and treatment.

Nomadic populations in Nigeria are often isolated and stigmatized, and reaching them with health services is difficult [9,10,30]. Several studies showed high rates of TB and other diseases among nomadic communities [9,10]. Other studies have shown low rates of knowledge about TB in different populations in Nigeria [31], and that knowledge was linked to education level. We also noted differences according to education levels. Despite being a survey conducted at schools, education and literacy rates are very low among nomads in Nigeria, and misconceptions about disease transmission and treatment were held by many respondents. Television, radio, newspapers, periodicals, direct counselling from medical staff, and the dispersion of information through local family and friend networks have been highlighted as sources of HIV/AIDS information [32]. While several studies have explored the role of media in influencing health behaviours, its specific impact on TB-related health-seeking behaviour is less extensively documented. However, a study in Colombia suggested that short-term impact on care-seeking can be achieved using information campaigns [33]. More evidence is needed to document how media can impact care-seeking behaviour for TB.

Through this targeted intervention, we detected TB rates of 494/100,000 population, almost twice the national estimates [1]. Nevertheless, our results are surely underestimated as we only used verbal screening and Xpert for testing, while more sensitive screening methods, especially those incorporating chest X-rays, would have diagnosed many more people with TB [34,35]. It is imperative to acknowledge that our decision to use verbal screening as a frontline approach was primarily driven by the context of our study population and logistical constraints. Being a hard-to-reach population with unique challenges, we aimed to employ a method that was both feasible and acceptable to the nomads, ensuring maximum participation. Moreover, we only identified 41 people with clinically diagnosed TB, whereas a more comprehensive survey using chest X-rays and other tests may have found many more people [36], including the subclinical/asymptomatic TB cases. 

There are several important limitations to our study. One of the limitations of this study is the absence of reliability and test–retest assessments to ensure internal consistency and consistency between the six researchers who conducted the interviews. The results of our KAP survey are not representative of the nomadic population, as they were not collected from a representative quantitative sample and we did not collect KAP survey data in a control population for comparison or conduct any internal comparison given the small sample size. We are also unable to define any direct relationship between the design of the ACF campaign and the intervention’s results as the study design did not provide the ability to compare interventions using the KAP results and a more generic ACF approach. Numerous ACF interventions in Nigeria and elsewhere have shown positive impact without an intervention designed by a KAP study [36,37,38], and the use of the KAP results must be interpreted in this light. However, the results show how meaningful engagement and an inclusive process can be used to develop people-centred interventions that can deliver equitable, acceptable, and quality health services to groups in need of services but that are often outside of routine care settings.

## 5. Conclusions

We demonstrated how the results of a KAP survey could be used to design a community-driven and -based TB and HIV case-finding intervention with and among a key population for TB in rural Nigeria, which identified high rates of TB in nomadic communities. Ensuring that the findings of behavioural and knowledge surveys are directly linked to programming is important to improve community engagement and rights-based approaches to TB and should be systematically part of TB care delivery interventions.

## Figures and Tables

**Table 1:** The parameters of age, sex, educational level, and occupation of the participants in the pre-intervention phase.

Socio-Demographic Characteristics	Nomadic Teachers (N = 36) (%)	Community Leaders (N = 18) (%)	Community Members (N = 27) (%)	Total (N = 81) (%)
Median Age				40.5 (IQR 34.5–44.5)
Gender				
Male	28 (77.8)	16 (88.9)	18 (66.7)	62 (76.5)
Female	8 (22.2)	2 (11.1)	9 (33.3)	19 (23.5)
Educational level				
None/some primary school	17 (47.2)	14 (77.8)	19 (70.4)	50 (61.7)
Completed primary/some high school	9 (25)	2(11.1)	3(11.1)	14(17.3)
Completed high school	6 (16.7)	0 (0)	3 (11.1)	9 (11.1)
Professional or degree	4 (11.1)	2 (11.1)	2 (7.4)	8 (9.9)
Occupation				
Cattle rearing	8 (22.2)	6 (33.3)	6 (22.2)	20 (24.7)
Crop farming	6 (16.7)	9 (50)	8 (29.6)	23 (28.4)
Government employment	16 (44.4)	1 (5.6)	3 (11.1)	20 (24.7)
Others	6 (16.7)	2 (11.1)	10 (37)	18 (22.2)

IQR = Interquartile range.

**Table 2:** Knowledge of TB and HIV/AIDS among nomads in Nigeria.

Knowledge Item	Nomadic Teachers (N = 36) (%)	Community Leaders (N = 18) (%)	Community Members (N = 27) (%)	Total (N = 81) (%)
Ever heard of tuberculosis (TB)? Yes	25 (69.4)	11 (61.1)	14 (51.9)	50 (61.7)
No	11 (30.6)	7 (38.9)	13 (48.1)	31 (38.3)
Cause of TB				
Evil spirit	5 (13.9)	1 (5.6)	4 (14.8)	10 (12.2)
Witchcraft	2 (5.6)	2 (11.1)	5 (18.5)	9 (11.1)
Food	6 (16.7)	5 (27.8)	3 (11.1)	14 (17.3)
Germs	15 (41.7)	7 (38.9)	4 (14.8)	26 (32.1)
Curse	3 (8.3)	0 (0)	2 (7.4)	5 (6.2)
Others	5 (13.9)	3 (16.7)	9 (33.3)	17 (21)
Signs of TB				
Fever	16 (44.4)	3 (16.7)	11 (40.7)	30 (37)
Weight loss	4 (11.1)	3 (16.7)	4 (14.8)	11 (13.6)
Cough	8 (22.2)	11 (61.1)	5 (18.5)	24 (29.6)
Sweating	2 (5.6)	0 (0)	2 (7.4)	4 (4.9)
Others	6 (16.7)	1 (5.6)	5 (18.5)	12 (14.8)
Have you ever heard of HIV? Yes	26 (72.2)	8 (44.4)	15 (55.6)	49 (60.5)
No/no response	10 (27.8)	10 (55.6)	12 (44.4)	32 (39.5)
What is your source of knowledge on HIV?				
Religious leaders	0 (0)	1 (5.6)	3 (11.1)	4 (4.9)
Health workers	18 (50)	7 (38.9)	10 (37)	35 (43.2)
Family and friends	1 (2.8)	4 (22.2)	2 (7.4)	7 (8.6)
Teachers	1 (2.8)	2 (11.1)	3 (11.1)	6 (7.4)
Posters and printed materials	0 (0)	0 (0)	1 (3.7)	1 (1.2)
Media (broadcasts)	12 (33.3)	4 (22.2)	4 (14.8)	20 (24.7)
Other	4 (11.1)	0 (0)	4 (14.8)	8 (9.9)
What is the relationship between HIV and TB?				
They are the same	5 (13.9)	4 (22.2)	6 (22.2)	15 (18.5)
They are not related	13 (36.1)	9 (50)	9 (33.3)	31 (38.3)
They complement each other	8 (22.2)	3 (16.7)	0 (0)	11 (13.6)
I do not know	4 (11.1)	0 (0)	4 (14.8)	8 (9.9)
How serious is HIV?				
Very serious	22 (61.1)	8 (44.4)	15 (55.6)	45 (55.6)
Somewhat serious	8 (22.2)	2 (11.1)	2 (7.4)	12 (14.8)
Not very serious	6 (16.7)	7 (38.9)	7 (25.9)	20 (24.7)
Can a person with HIV be cured? Yes	17 (47.2)	7 (38.9)	4 (14.8)	28 (34.6)
No/no Response	19 (52.8)	11 (61.1)	23 (85.2)	53 (65.4)

**Table 3:** Health care and information access among nomads in Nigeria.

Access Item	Nomadic Teachers (N = 36) (%)	Community Leaders (N = 18) (%)	Community Members (N = 27) (%)	Total (N = 81) (%)
Presence of a health facility in the community				
Yes	15 (41.7)	8 (44.4)	9 (33.3)	32 (39.5)
No/no response	21 (58.3)	10 (55.6)	18 (66.7)	49 (60.5)
Distance of the nearest health facility				
Less than 1 km	14 (38.9)	3 (16.7)	10 (37)	27 (33.3)
1–2 km	15 (41.7)	5 (27.8)	10 (37)	30 (37)
2–5 km	1 (2.8)	3 (16.7)	1 (3.7)	5 (6.2)
>5 km	6 (16.7)	4 (22.2)	2 (7.4)	12 (14.8)
Do not know	0 (0)	3 (16.7)	4 (14.8)	7 (8.6)
Type of nearest health facility				
Health center	27 (75)	14 (77.8)	20 (74.1)	61 (75.3)
Hospital	8 (22.2)	1 (5.6)	6 (22.2)	15 (18.5)
Private facility	1 (2.8)	3 (16.7)	1 (3.7)	5 (6.1)
Ever received any information about TB				
Yes	26 (72.2)	16 (88.9)	19 (70.4)	61 (75.3)
No/no response	10 (27.8)	2 (11.1)	5 (29.6)	20 (24.7)
Source of TB information				
Religious leaders	2 (5.6)	0 (0)	2 (7.4)	4 (4.9)
Health workers	16 (44.4)	9 (50)	9 (33.3)	34 (42)
Family and friends	4 (11.1)	1 (5.6)	4 (14.8)	9 (11.1)
Teachers	0 (0)	0 (0)	1 (3.7)	1 (1.2)
Media (broadcasts)	14 (38.9)	8 (44.4)	11 (40.7)	33 (40.7)
The most effective source of information				
Newspapers and magazines	1 (2.8)	2 (11.1)	4 (14.8)	7 (8.6)
Radio	11 (30.6)	6 (33.3)	7 (25.9)	24 (29.6)
TV	6 (16.7)	1 (5.6)	0 (0)	7 (8.6)
Billboards	2 (5.6)	0 (0)	0 (0)	2 (2.5)
Brochures, posters, and pamphlets	2 (5.6)	2 (11.1)	2 (7.4)	6 (7.4)
Health care workers	5 (13.9)	2 (11.1)	9 (33.3)	16 (19.8)
Family, friends, neighbours, and colleagues	6 (16.7)	5 (27.8)	3 (11.1)	14 (17.3)
Religious leaders	2 (5.6)	0 (0)	2 (7.4)	4 (4.9)
Teachers	1 (2.8)	0 (0)	0 (0)	1 (1.2)

**Table 4:** TB and HIV screening among nomadic schools and communities in Adamawa.

TB Screening Cascade	Female	Male	Total
Nomadic school and community screening events for TB	-	-	267
Individuals verbally screened for TB	30,535	31,356	61,891
Individuals with presumptive TB (% of screened)	2640	2453	5093 (8.2%)
People with presumptive TB tested by Xpert (% of presumptive)	2364	2183	4547 (88%)
Individuals with bacteriologically confirmed TB detected and treated (% of tested)	78	147	225 (4.9%)
Individuals with all forms of TB detected and treated (% of presumptive)	158	148	306 (6.0%)
Individuals counselled and tested for HIV (% of presumptive)	2352	2137	4489 (88%)
Individuals testing positive for HIV (% of tested for HIV)	21	48	69 (1.5%)

## Data Availability

Study data can be made available from the Adamawa State Ministry of Health, admoh_yola@yahoo.com, upon request.

## Supplementary Materials

The following supporting information can be downloaded at: https://www.mdpi.com/article/10.3390/tropicalmed9080167/s1, File S1: A Questionnaire to Explore TB Related Knowledge Attitudes and Practices.
