# Supplementary material for: Using a Knowledge and Awareness Survey to Engage and Inform a Community-Based Tuberculosis Intervention among Nomads in Adamawa State, Nigeria
Source: Trop Med Infect Dis. 2024 Jul 23;9(8):167. doi: 10.3390/tropicalmed9080167 (PMC11359801; doi:10.3390/tropicalmed9080167)
Supplement: Supplementary file 1 [file tropicalmed-09-00167-s001.zip › tropicalmed-2967693-supplementary.pdf]

A QUESTIONNAIRE TO EXPLORE TB RELATED KNOWLEDGE ATTITUDES AND PRACTICES

LGA:…………………….. Community:…………………….. Type of Respondent: Teacher/Comm Leader/Mem
Code:……………… Date: …../……./2018

We wish to learn about your Knowledge Attitudes and Practices regarding Tuberculosis. (TB). We hope to understand your needs and the best way to bring information to you as well as understand the barriers you may face in seeking treatment for TB.
Your answers will not be released to anyone but will only be used for the purpose stated above. In this light your name will not be written on the questionnaire or kept in any other records. Your participation in this exercise is voluntary, however we would be grateful if you participated as we would like to collect as much information as possible. It is anticipated that this exercise will take about an hour to complete.

Name of interviewer..................................
Date: ……/……../2018

A: GENERAL AND DEMOGRAPHIC QUESTIONS (*Please Tick Appropriately)*

1 How old are you? (Age as at last birth day)

a.	Under 15	
b.	15 – 24	
c.	25 – 34	
d.	35 – 44	
e.	45 – 54	
f.	55 – 64	
g.	65 and above	

2 Gender:

a.	Male	
b.	Female	

3 What is the highest level of education that you have completed?

a.	No Schooling at all	
b.	Literacy classes only	
c.	Some Primary School	
d.	Completed Primary School	
e.	Some High School	
f.	Completed High School	
g.	Higher education after completing High School (professional or Degree).	

4 What work do you do to earn a living?

a.	Cattle rearing	
b.	Crop Farming	
c.	Government Employment	
d.	Others; Pls specify……..	

5 Is there a health facility in your community?

a.	Yes	
b.	No	

6 If yes, how far is the nearest health facility?

a.	<1 Km	
b.	1 – 2 Km	
c.	2 – 5 Km	
d.	>5 Km	
e.	Don't Know	

7 What type is the facility?

a.	Health Center	
b.	Hospital	
c.	Private Facility	
d.	Others	

B: Knowledge

i. Have you ever heard of Tuberculosis (TB)?

	TB	
a.	Yes	
b.	No	

ii. What do you think is the cause of TB? *(Please tick all that applies)*

a.	Evil spirit	
b.	Witchcraft	
c.	Food	
d.	Germs	
e.	Course	
f.	Others: Specify	

iii. What are the signs of Tuberculosis? *(Please tick all that applies)*

a.	Fever	
b.	Weight Loss	
c.	Cough	
d.	Sweating	
e.	Others: Specify	

iv. Have you ever received any information about TB?

a.	Yes	
b.	No	

v. If Yes, where did you learn about TB? *(Please tick all that applies)*

a.	Religious Leaders	
b.	Health Workers	
c.	Family and Friends	
d.	Teachers	
e.	Posters and printed materials	
f.	Media (broadcasts)	
g.	Others: specisy	

vi. What are the sources of information that you think can most effectively reach people like you with information on TB? *(Please tick all that applies)*

a.	Newspapers & magazines	
b.	Radio	
c.	TV	
d.	Billboards	
e.	Brochures, posters and pamphlets	
f.	Health Care Workers	
g.	Family, friends, neighbors and colleagues	
h.	Religious leaders	
i.	Teachers	
j	Other; specify	

vii. What are the signs and symptoms of TB that you know *(Please tick all that applies)*?

a.	Rash	
b.	Cough	
c.	Cough lasting longer than 3 weeks	
d.	Coughing up blood	
e.	Severe headache	
f.	Nausea	
g.	Weight loss	
h.	Fever	
i.	Fever without clear cause that lasts more than days	
j.	Chest pain	
k.	Shortness of breath	
l.	Ongoing fatigue	
m.	Do not Know	
n.	Other; specify	

viii. How can a person get TB *(Please tick all that applies)*?

a.	Through hand shakes	
b.	Through the air when a person with TB coughs or sneezes	
c.	Through sharing dishes	
d.	Through eating from the same plate	
e.	Through touching items in public places: door knobs, handles, in public transport	
f.	Do not know	
g.	Others; specify: ………….	

ix. How can a person prevent getting TB *(Please tick all that applies)*?

a.	Avoid shaking hands	
b.	Cover mouth and nose when coughing	
c.	Avoid sharing dishes	
d.	Washing hands after touching items in public places.	
e.	Closing windows at home	
f.	Good nutrition	
g.	Through prayer	
h.	Do not know	
i.	Others; specify	

x. In your opinion, who can be infected with TB? *(Please tick all that applies)*?

a.	Anybody	
b.	Only poor people	
c.	Only homeless people	
d.	Only alcoholics	
e.	Only drug users	
f.	Only people living with HIV	
g.	Only people who have been in prison	
h.	Others: specify……..	

xi. Can TB be cured?

a.	Yes	
b.	No	

xii. If Yes, how can someone with TB be cured? *(Please tick all that applies)*

a.	Drugs given by a health center	
b.	DOTS	
c.	Herbal remedies	
d.	Specific drugs given by a health center	
e.	Home rest without medicine	
f.	Traditional medicine	
g.	Faith healing through prayer	
h.	I do not know	
i.	Other	

xiii. Do you feel well informed about TB?

a.	Yes	
b.	No	

xiv. Do you wish to get more information about TB?

a.	Yes	
b.	No	

xv. Do you think you can get TB?

a.	Yes	Because………………………………………………………………………………………… …………………………………………………………………………………………………….
b.	No	Because………………………………………………………………………………………… …………………………………………………………………………………………………….

xvi. How expensive do you think TB diagnosis and treatment is in the Country?

a.	It is free of charge	
b.	It is reasonably priced	
c.	It is moderately /somewhat expensive	
d.	It is very expensive	

Knowledge on HIV and other Co-Morbid Conditions

i. Have you ever heard of HIV?

a.	Yes	
b.	No	

ii. If Yes, what is the relationship with TB? *(Please tick all that applies)*

a.	They are the same	
b.	They are not related	
c.	They complement each other	
d.	I don't know	
e.	Others: Specify…………	

iii. If Yes, where did you learn about HIV? *(Please tick all that applies)*

a.	Religious Leaders	
b.	Health Workers	
c.	Family and Friends	
d.	Teachers	
e.	Posters and printed materials	
f.	Media (broadcasts)	
g.	Others: specify	

iv. Can you mention any disease that is related to TB? *(Please tick all that applies)*

a.	Diabetes	
b.	HIV	
c.	Hepatitis	
d.	Malaria	
e.	Diarrhea	
f.	Polio	
g.	Others; Specify…………	

v. Can a person with HIV be cured?

a.	Yes	
b.	No	

B: Attitude to TB

i. What is your perception of the disease Tuberculosis?

a.	Nothing	
b.	Serious	
c.	Moderate	
d.	Others: Specify	

ii. Do you know people who have TB/ had TB?

a.	Yes	
b.	No	

iii. Which statement is closest to you about people with TB disease?

a.	I feel compassion and desire to help	
b.	I feel compassion but I tend to stay away from these people	
c.	It is their problem and I cannot get TB	
d.	I fear them because they may infect me	
e.	I have no particular feeling	
f.	Others; Explain…………	

iv. In your community, how is a person who has TB usually regarded/ treated?

a.	Many people reject him/her	
b.	Most people are friendly but they generally try to avoid him/her	
c.	The community mostly supports and helps him/her	
d.	Other, please explain..............................	

v. What would your reaction be if you found out that you had TB? *(Please tick all that applies)*

a.	Fear	
b.	Surprise	
c.	Shame	
d.	Embarrassment	
e.	Sadness and hopelessness	
f.	Other, please explain............................	

vi. If you had TB, who would you talk to about your illness? *(Please tick all that applies)*

a.	No one	
b.	Health staff	
c.	Spouse	
d.	Children	
e.	Parents	
f.	Close friend	
j	Other, specify..........................	

Attitude on HIV and other Co-Morbid Conditions

i. In your opinion, how serious is HIV?

a.	Very Serious	
b.	Somewhat Serious	
c.	Not Very Serious	

ii. How serious a problem is HIV in your community?

a.	Very Serious	
b.	Somewhat Serious	
c.	Not Very Serious	

Practices:

i. Where do you usually go when you are sick or to treat a general problem?

a.	Government Clinic/Hospital	
b.	Private Clinic/Hospital	
c.	Traditional Healer	
d.	Others: Specify……	

ii. How often do you generally seek health care at a clinic or hospital?

a.	Twice or more a year	
b.	Once a year	
c.	Once every 2 years	
d.	Once or twice every 5 years	
e.	Never in the last 5 years	
f.	Others; explain……..	

iii. In your opinion, how serious is TB?

a.	Very Serious	
b.	Somewhat Serious	
c.	Not Very Serious	

iv. How serious a problem is TB in your community?

a.	Very Serious	
b.	Somewhat Serious	
c.	Not Very Serious	

v. What will you do if you thought you have TB? *(Please tick all that applies)*

a.	Go to a health facility	
b.	Go to a pharmacy	
c.	Go to a traditional healer	
d.	Pursue other self-treatment options	
e.	Others; Specify….	

vi. If you had symptoms of TB, at what point would you seek help from a health facility?

a.	As soon as I realize that the symptoms might be related to TB	
b.	When self-treatment does not work	
c.	When the TB symptoms have lasted 3-4 weeks	
d.	I would not go to a doctor or health facility	

vii. If you would not go to a facility, what is the reason? *(Please tick all that applies)*

a.	Not sure where to go	
b.	Cost	
c.	Transport challenges/ distance from health facility	
d.	Do not trust health workers	

e.	Do not like the attitude of Health workers	
f.	Cannot leave work, health facility operation hours not convenient/ clash with work hours	
g.	Fear to find out that something is really wrong.	
h.	Others; Explain………..	

viii. Would you encourage a suspected TB patient to seek treatment?

a.	Yes	
b.	No	

ix. If No, what is (are) your reason?

a.	Is not my business	
b.	Fear of infecting others	
c.	There is no need	
d.	Cost of treatment	
e.	Distance of treatment place	
f.	I don't know where to refer	
e.	Others; Specify…………	

x. Is there anything else you would like to share with me regarding Tuberculosis/TB?

……………………………………………………………………………………………..
……………………………………………………………………………………………..
……………………………………………………………………………………………..

Thank you for your participation in this survey

Quality check:

	Name	Signature & Date
Questionnaire administered by:		
Completeness and consistency checked by:		
Categorization/ coding done by:		
Entered in data master sheet by:		
Countersigned by team leader:		
